# A preliminary study of hardness and modulus of elasticity in sheep mandibles submitted to distraction osteogenesis and low-level laser therapy

**DOI:** 10.4317/medoral.17280

**Published:** 2011-07-15

**Authors:** Angelo-Luiz Freddo, Roberto Hübler, Carlos-Afonso de Castro-Beck, Cláiton Heitz, Marília-Gerhardt de Oliveira

**Affiliations:** 1 MSc, Oral and Maxillofacial Surgery, School of Dentistry, Pontifícia Universidade Católica do Rio Grande do Sul (PUCRS), Porto Alegre, RS, Brazil. Research grant holder, Conselho Nacional de Desenvolvimento Científico e Tecnológico (CNPq); 2 PhD, Professor, School of Physics, PUCRS, Porto Alegre, RS, Brazil. Productivity grant holder, CNPq; 3 PhD, Professor, School of Veterinary Medicine, Universidade Federal do Rio Grande do Sul (UFRGS), Porto Alegre, RS, Brazil; 4 PhD, Professor, School of Dentistry, PUCRS, Porto Alegre, RS, Brazil; 5 PhD, Professor, School of Dentistry, PUCRS, Porto Alegre, RS, Brazil. Productivity grant holder, CNPq

## Abstract

Objectives: To investigate the quality of newly formed bone in sheep mandibles submitted to distraction osteogenesis and low-level laser therapy (LLLT), based on hardness and modulus of elasticity values. The ideal moment for laser application (during the latency/activation period vs. during the bone consolidation period) was also evaluated. Computed tomography imaging was used to assess relapse as a result of early device removal. Study design: Extraoral distraction devices were placed in five sheep so as to achieve 1.5 cm of lengthened bone in 60 days. Distraction devices were removed 50, 40, and 33 days after surgery. Four animals were treated with LLLT, at different times, and one was used as control (no LLLT). Results: When applied during the bone consolidation period, LLLT caused an increase in hardness and modulus of elasticity values. On the other hand, animals irradiated with LLLT during the latency/activation period presented a delay in bone healing. A period of consolidation of 13 days (early device removal) was associated with relapse. Conclusions: Nanoindentation tests were able to detect slight abnormalities in bone metabolism and proved to be important tools for the assessment of bone quality following distraction osteogenesis. LLLT provided increased benefits when applied during the bone consolidation period, once it promoted an increase in hardness and modulus of elasticity values. According to our results, the bone consolidation period should be of at least 3 weeks, so as to prevent relapse.

** Key words:** Osteogenesis distraction, low-level laser therapy, elastic modulus, hardness tests.

## Introduction

Distraction osteogenesis has become an increasingly consolidated alternative method for facial bone reconstruction, with promising results. It can be used to correct congenital defects, defects caused by trauma, after oncological surgeries and for the oral rehabilitation of patients with osteointegrated implants. However, the long-term stability of results obtained with distraction osteogenesis is not well documented, and reports of instability and relapse can be found in the literature (1). Therefore, the main focus of current studies in the field of distraction osteogenesis has been to accelerate the bone maturation process and to improve the physical properties of lengthened bones (1).

In this sense, low-level laser therapy (LLLT) is a procedure that uses large portions of the visible and infrared light spectra to improve the healing process by stimulating vascularization, fibroblast proliferation and the deposition of collagen (2,3). The effects of the photochemical and photobiological properties of lasers on the biomodulation of inflammatory and bone repair processes has been studied with the aims of accelerating bone healing, decreasing postoperative discomfort and edema and improving tissue regeneration in patients submitted to surgery (4,5).

Miloro et al. (6) have conducted a study to assess whether irradiation with LLLT (GaAlAs) would accelerate bone regeneration and reduce the bone consolidation period in distracted rabbit mandibles. Based on the study results, the authors concluded that the application of laser during the bone consolidation period accelerates the bone regeneration process and allows for earlier removal of devices, thus reducing morbidity.

New bone formation can be assessed using several tests, of variable complexity and accuracy. Nanoindentation tests are able to reveal physical properties such as hardness, apparent modulus of elasticity, defects, and residual stresses in the areas analyzed (7). Among these variables, hardness and modulus of elasticity are useful to identify the quality of newly formed bone, as well as changes in the metabolism of bone formation. 

The objective of the present study was to assess the quality of newly formed bone in sheep mandibles submitted to distraction osteogenesis and LLLT, based on hardness and modulus of elasticity values. In addition, the present study aimed to assess the ideal moment for laser application, whether during the latency/activation period or during the bone consolidation period. Finally, the study also assessed relapse after early device removal, based on computed tomography (CT) findings. 

## Material and Methods

The present research protocol was approved by the Research Ethics Committee at Pontifícia Universidade Católica do Rio Grande do Sul (PUCRS), Porto Alegre, RS, Brazil. Five female Corriedale sheep, aged 2 years and weighing 40-45 kg, were included in the investigation following basic laboratory tests conducted to discard the possible presence of any diseases prior to surgery that could interfere with the study results.

The animals (sheep A, B, C, D, and E) were submitted to the same distraction osteogenesis protocol and assessed for a period of 60 days. In sheep A, the distraction device was used for 50 days, in sheep B for 40 days, and in sheep C, D, and E for 33 days. LLLT employed the same parameters in all sheep, and was applied during the latency/activation period in animals A, B, and C, and during the bone consolidation period in sheep D; sheep E was not submitted to LLLT. 

Throughout the study period, the animals were housed in pairs, in appropriate cages, at the animal hospital of Universidade Federal do Rio Grande do Sul, Porto Alegre, RS, Brazil. Each animal was identified with a plate displaying the corresponding letter. 

Anesthesia procedures were carried out by a veterinarian involved in all phases of the study, both prior to surgery, during the procedures, and after surgery. General anesthesia was induced with acepromazine 0.05 mg/kg, meperidine 2 mg/kg, ketamine 4 mg/kg, and halothane mask. Propofol 8 mL was used during intubation. Anesthesia was maintained with halothane in 100% oxygen (O2). Additional, maintenance intramuscular doses were administered as necessary. Antibiotic prophylaxis was performed with intravenous administration of ampicillin sodium 10 mg/kg.

Surgery was then initiated, as follows: trichotomy of the left submandibular region, antisepsis, local infiltration of 3 mL of 1% lidocaine and epinephrine 1:100.000, and incision (Risdon type) 1 cm below the left basilar region of the mandible, measuring approximately 3 cm. Using a layered approach, tissues were separated through both the lateral and medial faces of the mandible by blunt dissection using Metzenbaum scissors and detachers. Farabeuf retractors were positioned to expose the lateral surface of the mandible. Corticotomies were performed with a reciprocating saw through the medial and lateral faces of the mandible, in the mandibular angle region. The distraction device was fixed with four transcutaneous screws under irrigation with saline solution. Distractors were then activated until encountering resistance, and fractures were produced with straight chisels. The surgical wound was closed in layers with single interrupted sutures using 4.0 monofilament nylon (8). 

After surgery, the sheep remained in the animal hospital and received morphine 0.4 mg/kg every 8 hours for a total of 72 hours, in addition to pentabiotics (benzathine penicillin, procaine, and streptomycin combined) every 24 hours, for 7 days. 

The device used in laser irradiation was Thera Laser® (DMC, São Carlos, Brazil) with gallium-aluminum-arsenide (GaAlAs) active medium, wavelength of 830 ηm (infrared), properly calibrated. 

The sheep received laser doses directly on the distraction site, at three points across the osteotomy area, with 5 J/cm2 applied to each point, totaling 15 J/cm2, with a power of 50 mW, in a continuous wave mode, for 1.41 minutes. The total energy applied at the end of the experiment amounted to 120 J/cm2. In sheep A, B, and C, submitted to LLLT during the latency/activation period, irradiation was first applied immediately after closure of the surgical wound and then every 48 hours, at a total of eight sessions. In sheep D, submitted to LLLT during the bone consolidation period, the first irradiation session took place after the last activation, and then every 48 hours, also at a total of eight sessions. Sheep E (control) was not submitted to LLLT. Because laser therapy is painless, no sedation or anesthetics were necessary. 

 -Distraction osteogenesis protocol

 Latency period – 5 days (days 1 to 5): the distraction device was not activated; it was only inspected and cleaned with 1% iodophor alcohol. 

 Activation period – 15 days (days 6 to 20): device activation started on the sixth postoperative day at a rate of 1 mm per day, to a total of 15 mm at the end of the activation period.

 Bone consolidation period – 13 to 30 days (days 21 to 33, 40, or 50): after the activation period, the distractor remained in sheep A for 30 days, in sheep B for 20 days, and in sheep C, D, and E for 13 days, working as a rigid fixation device, so that bone maturation was achieved. Following the bone consolidation period assigned to each animal, distractor removal was carried out under local anesthesia.

Sixty days after surgery, all five animals were killed under deep anesthesia, due to cardiorespiratory arrest, as recommended by the Universal Declaration on Animal Welfare. Once death was confirmed by the absence of vital signs, mandibles were dissected and stored in glutaraldehyde, and subsequently submitted to CT imaging. Images were prepared and analyzed in the Laboratory of Materials and Nanosciences, Study Group on Properties of Surfaces and Interfaces, Center for Physics Research and Development, School of Physics, PUCRS (8).

**Figure 1:**
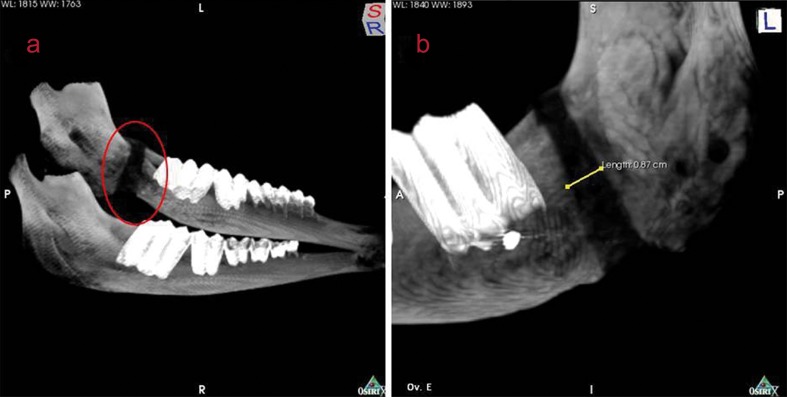
A) Three-dimensional, maximal intensity projection reconstructions of computed tomography images and B) linear measure between the edges of mature bone.

The amount of lengthened bone was assessed using CT images obtained with a Somaton Plus 4 scanner (Siemens®, Berlin, Germany). Samples were positioned so as to allow the production of axial 0.5 mm-thick sections. The PixWiwer® software (Florianópolis, Brazil) was used for image manipulation and for the production of three-dimensional maximal intensity projection (MIP) reconstructions (Fig. 1.A).

MIP images were used to measure the bone gap, as a linear measure between the edges of mature bone (new, immature bone formed by distraction osteogenesis shows hypodensity on CT). Two examiners were responsible for the measurements obtained in each reconstruction, in both axial and coronal directions (Fig 1.B).

In order to compare the influence of time of device use on the quality of newly formed bone, the following total times were allowed for each animal: sheep A, 50 days; sheep B, 40 days; and sheep C, D, and E, 33 days. 

Following CT, the mandibles of each animal were sectioned in two halves, the operated side and the control side (non-operated). Specimens were embedded in acrylic resin and sectioned in axial direction in three blocks using a band saw. All blocks were properly identified as control or operated side, and as upper, middle or lower portion.

In order to obtain perfectly plane and polished surfaces, as demanded by the nanoindentatoin tests employed, bone blocks were submitted to a polishing sequence using eight water-cooled sandpapers (80, 150, 320, 400, 600, 1200, 2400 e 4000); at each interval (sandpaper change), specimens were immersed in fully deionized water, using an ultrasound device, for approximately 5 minutes. Specimens were polished with sandpapers 600, 1200, 2400, and 4000 using a 1:1 mixture of propylene glycol and isopropyl alcohol, and dried with nitrogen gas. At the end of the polishing process, specimens were finished with 9 μm, 1 μm, and 1/4 μm diamond polishing paste (DPPaste; StruersTM), and finally with silica carbonate solution and water using a metallographic polishing machine (8). 

**Figure 2:**
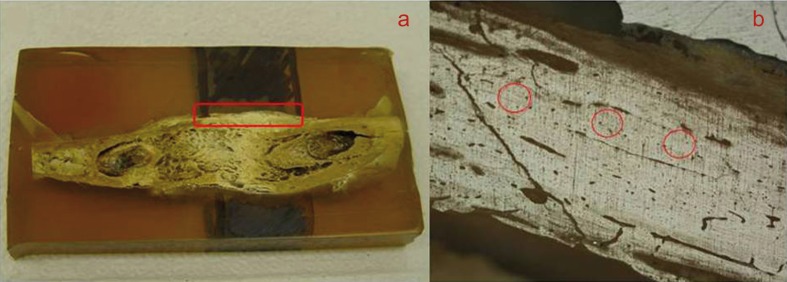
A) Polished bone block; red square showing the external cortical bone area assessed by nanoindentation B) Red circles indicating areas of the external cortical bone assessed by nanoindentation (optical photomicrographs at 50x magnification).

Specimens were analyzed and photographed using an optical microscope (Olympus BX 60TM) at 50 x magnification in order to identify the elongated bone area and to define the regions to be assessed. The photomicrographs obtained were marked with red circles indicating reference points, i.e., regions to be measured in the nanoindentation tests, always on the external cortical bone (Fig.2.A, B). The physical properties of newly formed bone were analyzed based on the variables hardness and modulus of elasticity (nanoindentation test – Oliver-Pharr method), using a hardness indenter with Berkovich geometry and a dynamically controlled device (HV100, Fischerscope, Helmut-Fischer Inc., Stuttgart, Germany). The following parameters were standardized in the analysis: use of a 50 mN load to assess the polished samples; cycles run at intervals of 40 seconds loaded; a holding time of 30 seconds at maximum load (50mN, Pmax), so as to minimize the effects of the experiment in terms of viscoelastic deformation of the bone; 40 seconds unloaded (from 50 mN to zero). Measurements were obtained along a line over the external cortical bone, from 0.5 mm before up to 0.5 mm beyond the region of newly formed bone, at a total of 34 measurements per sample. On the control side of each animal, measurements were carried out on the external cortical bone, at a total of six measurements per sample, in view of the similarity observed across the samples. The objective of obtaining several measurements was to assess the whole extension of the distracted area, once different levels of bone maturation were found across the bone tissue, as a result of the gradual lengthening process that is characteristic of distraction osteogenesis.

## Results

**Figure 3:**
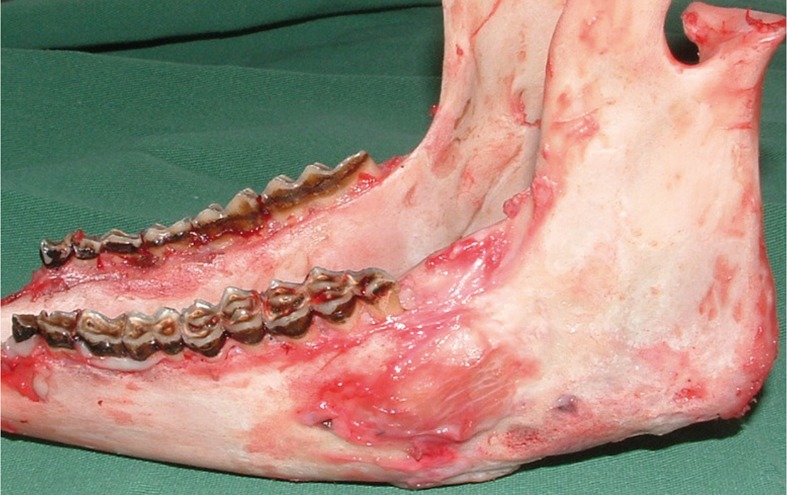
Macroscopic analysis of mandible.

The macroscopic analysis carried out during mandible dissection revealed the presence of a bone callus in the distracted region of all specimens, with different degrees of mineralization on the cortical bone (Fig. 3).

CT images were used to measure the distracted bone region and to assess the influence of early device removal on bone expansion results. The three-dimensional MIP images showed differences in bone density, with a distraction distance (gap) of 14.9 mm for sheep A and 13.7 mm for sheep B. Sheep C, D, and E, submitted to only 13 days of bone consolidation, presented relapse, with a mean gap of 9.9 mm, 9.9 mm, and 8.8 mm, respectively.

Tables 1 and 2 show mean and standard deviation obtained for nanohardness and modulus of elasticity in each specimen, on both the operated and control sides, as a result of the different distraction and LLLT protocols employed. The application of LLLT during the bone consolidation period caused an increase in hardness and modulus of elasticity values, compared to a delay in bone healing in specimens submitted to LLLT during the latency/activation period.

## Discussion

The distraction osteogenesis protocol adopted in the present study, namely, a latency period of 5 days and device activation once a day at 1 mm/day, is widely used and consolidated in the literature (9-12). The choice of a consensual distraction protocol is justified by our main objective of assessing the influence of laser on the quality of newly formed bone.

Postoperative time and the decision to keep animals alive for 60 days was based on the need for a minimum amount of time to allow the assessment of relapses. Moreover, several previous studies had already adopted the same period of 60 days to assess bone healing, e.g. Friesen et al. (13) and Pinheiro et al. (14).

Distraction osteogenesis involves metabolic activities that are potentially biomodulated by the use of laser. Therefore, laser therapy is believed to accelerate the healing process in newly formed bone, thus reducing the overall treatment time.

Current developments of the distraction osteogenesis technique lie essentially in removing the distractor as early as possible, whenever bone quality and dimensional stability are observed. In our study, sheep A used the inactivated distractor for 30 days, and the CT images revealed relapse, with a contraction of 0.1 mm in the distracted area in that animal. Sheep B showed slight dimensional instability after 20 days of bone consolidation, with a contraction of 1.3 mm in the distracted bone area (gap of 13.7 mm on CT). This difference observed in sheep B, however, might be the result of a bias in the distraction technique, once activations were performed manually. On the other hand, sheep C, D, and E, in which the bone consolidation period was significantly reduced to only 13 days, as a result of early device removal, showed a contraction of 5-6 mm in the distracted area. In addition, all three animals submitted to a bone consolidation period of 13 days, either treated with laser or not, presented relapse. These findings are in agreement with other published studies (15, 16), which have recommended a bone consolidation period between 3 and 7 weeks. Our results indicate that 3 to 4 weeks are enough to guarantee some degree of bone consolidation, with no risks of relapse and no need to extend this uncomfortable part of the treatment; this recommendation is corroborated by Zheng and Cheung (17). 

The present study showed that laser application yielded positive results when applied during the bone consolidation period. On the other hand, when applied during the latency and activation period, laser therapy caused a decrease in hardness and modulus of elasticity values, with a consequent delay in bone calcification.

The results obtained in sheep D (LLLT applied during the bone consolidation period), namely hardness of 414.4 MPa and modulus of elasticity of 12.3 GPa ( Table 1), are compatible with the results described by Cerqueira et al. (18) and Kreisner et al. (19), who also reported positive results in terms of bone regeneration and new bone formation in mandibles submitted to distraction osteogenesis and LLLT in the bone consolidation period.

Sheep E (control) was not submitted to LLLT. Although this sheep had a shorter bone consolidation period when compared with sheep A and B, the physical properties observed in the mandible of sheep E were higher than those observed for all sheep treated with LLLT during the activation period. The lowest modulus of elasticity and hardness results were found in sheep C, which used the distractor for 33 days and was submitted to LLLT in the activation period. These findings may suggest that the application of LLLT during the activation period in fact provokes a delay in bone healing, a hypothesis that is reinforced by the marked presence of cartilage tissue and endochondral ossification observed in specimens irradiated with LLLT during the activation period, as proposed by Cerqueira et al. (18).

Although the use of distraction osteogenesis has become increasingly common, it can cause significant discomfort to the patient, especially when external devices are used, possibly leading to infections, paresthesia, hypertrophic scars and social difficulties (14). Therefore, when this clinical and surgical approach is used to treat humans, early device removal becomes especially important. Taking into consideration that hardness and modulus of elasticity proved to be highly precise in the assessment of bone quality, new studies should be carried out, with larger animal samples, to assess these properties in newly formed bone submitted to different laser therapy protocols.

**Table 1:**
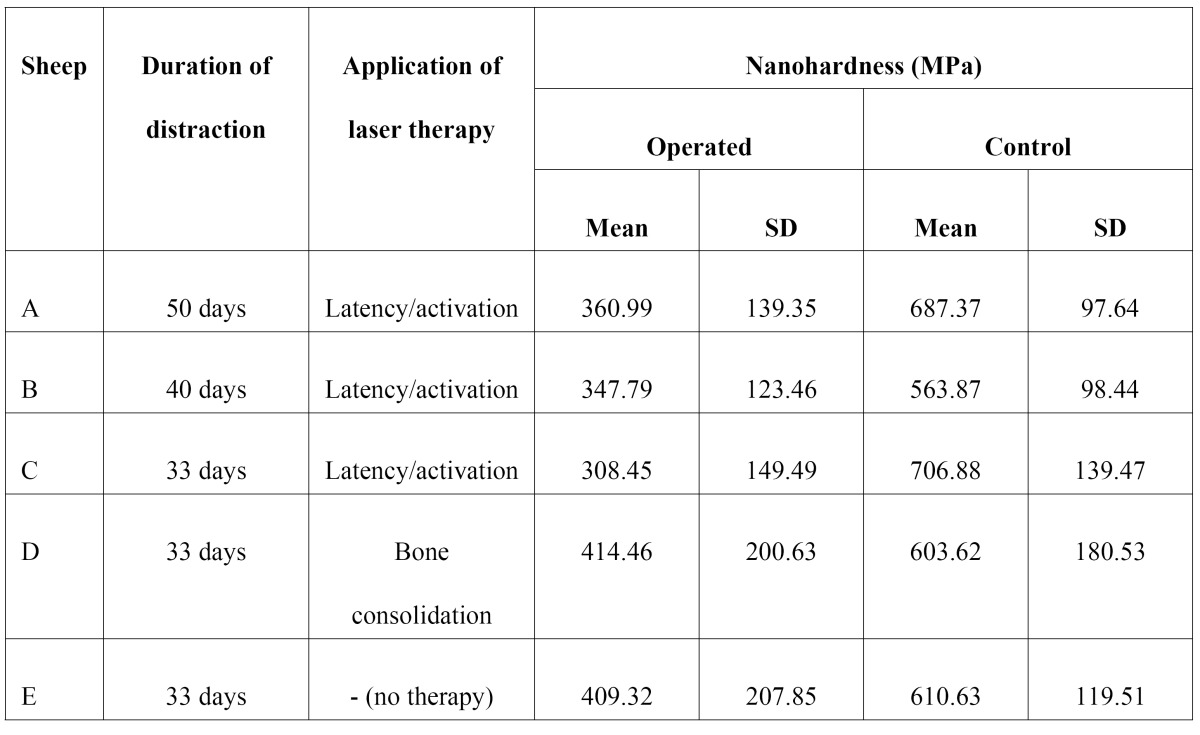
Mean and standard deviation values obtained for nanohardness.

## Conclusion

LLLT provided increased benefits when applied during the bone consolidation period, with relevant increases in hardness and modulus of elasticity values. A bone consolidation period of at least 3 weeks should be allowed so as to prevent relapse. ( Table 2)

**Table 2:**
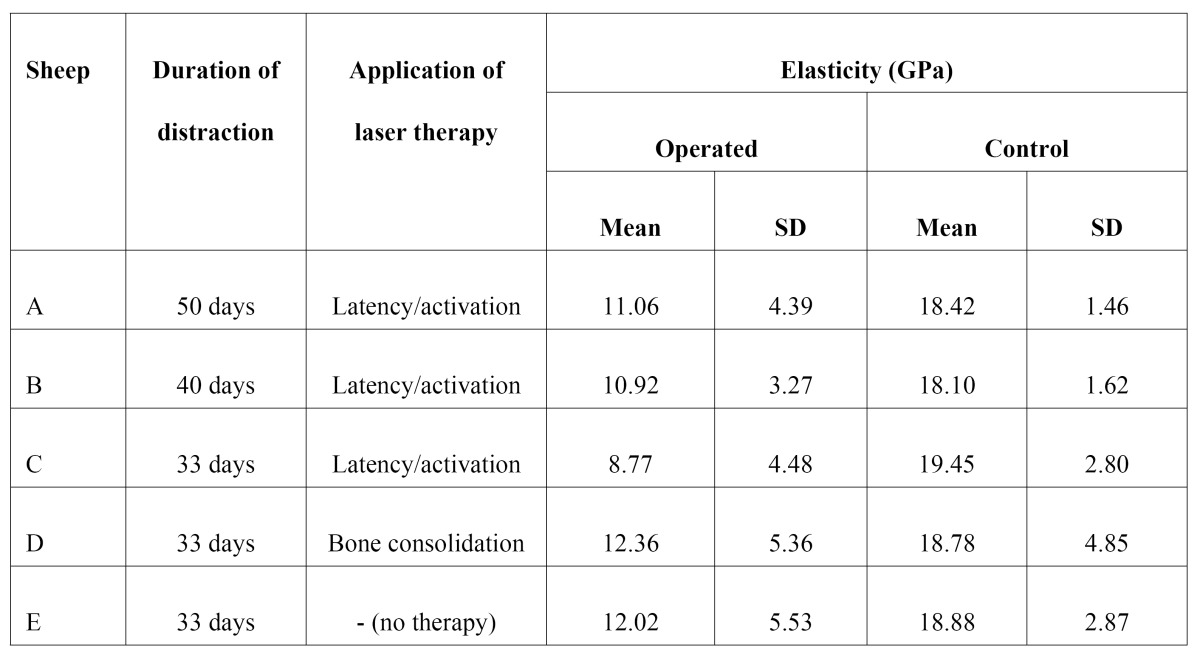
Mean and standard deviation values obtained for modulus of elasticity.
